# Mechanical ventilation and neurocritical patients: is there a role for anti-neuroinflammatory therapies?

**DOI:** 10.1186/s13054-020-2737-6

**Published:** 2020-01-22

**Authors:** Giovanni Giordano, Francesco Pugliese, Federico Bilotta

**Affiliations:** Department of Anaesthesia and Intensive Care, University La Sapienza, Rome, Italy

Dear Editor,

We read with great attention and interest the review by Robba and colleagues on mechanical ventilation (MV) in patients with acute ischemic stroke [1].

The authors examined the pathophysiology of stroke and the risk for pulmonary complications (brain-lung “dangerous” crosstalk, immunological response after stroke, stroke-associated pneumonia, and dysphagia) then concluding with useful recommendations on optimal ventilator settings and therapeutic strategies.

Several preclinical evidence supports that MV correlates with neuroinflammation and cognitive dysfunction [2, 3].

Surprisingly the authors in their review cite a paper from Hegeman and colleagues that challenge the hypothesis of the relationship between MV and brain inflammation state: “In the brain, MV did not induce a significant change in adhesion molecule mRNA expression as compared with non-ventilated controls, […] did not induce a detectable cytokine or chemokine response, [...] myeloperoxidase activity was below detection level in all experimental groups” [4].

Of interest, the study from Klinger and colleagues reported evidence on the effect of intravenous lidocaine on the transcerebral inflammatory response during cardiac surgery [5]. In their randomized controlled clinical trial, the authors aimed to investigate the potential anti-neuroinflammatory effect of intravenous lidocaine and observed “a reduction in the transcerebral activation of platelet-monocyte conjugates after aortic cross-clamp release. This may be a manifestation of reduced cerebral inflammation during cardiopulmonary bypass in response to treatment with lidocaine”.

To conclude, do the authors, according to the collected evidence, consider it relevant to the neuroinflammation associated with MV?

Do the authors consider it worthy—in patients with acute brain damage—to establish an appropriate anti-inflammatory therapy for preventing additional iatrogenic damage, for example, by taking advantage of the blunting effect of lidocaine on neuroinflammatory response?

## Authors’ response

Battaglini D; Bonatti G; Robba C; Rocco PRM; Pelosi P

Dear Editor,

We thank Professor Bilotta and colleagues for their interest in our recent scientific contribution [1]. The authors pointed out the role of neuroinflammation due to mechanical ventilation in stroke, thus advising the relevance of possible novel pharmacological strategies.

First, we were asked to clarify our point of view regarding the existence of significant neuroinflammation induced by mechanical ventilation in stroke, as challenged by Hegeman and colleagues [4]. We fully agree with the importance of neuroinflammation due to mechanical ventilation in stroke patients, and in our review, discussed only few of those papers referring to neuromodulation in stroke. Different perspectives have been reported in the literature, which focus on the complex interaction among the neuroendocrine, neuroinflammatory, autonomic and immunologic pathways, both implicated in lung injury that can affect the brain [6]. Furthermore, different neuromodulation patterns, as well as different brain-lung interactions, may occur due to mechanical ventilation or primary cerebral pathologies.

Second, as suggested, novel anti-inflammatory therapies for modulating the neuroinflammatory response (such as lidocaine) could play a role in the near future. This is another interesting issue that we did not take into consideration in our review, which focused on mechanical ventilation strategies in stroke. Experimental and clinical perspectives suggest a wide range of promising therapies against neuroinflammation in stroke, including molecular modulation, such as those reported by the use of minocycline in rats [7] or by modulating the vagal and dopaminergic pathways [8]. Newer biomolecular targets, such as noradrenergic and dopaminergic receptors, stem cell therapy, microRNA, and interleukins, all of which are still in the preclinical stages of research, are also of the utmost interest. However, addressing these would have exceeded the scope of our study.

## Data Availability

Not applicable.

## References

[1] Robba C, Bonatti G, Battaglini D, Rocco PRM, Pelosi P (2019). Mechanical ventilation in patients with acute ischaemic stroke: from pathophysiology to clinical practice. Crit Care.

[2] Kamuf J, Garcia-Bardon A, Ziebart A (2018). Lung injury does not aggravate mechanical ventilation-induced early cerebral inflammation or apoptosis in an animal model. PLoS One.

[3] Bilotta F, Giordano G, Sergi PG, Pugliese F (2019). Harmful effects of mechanical ventilation on neurocognitive functions. Crit Care.

[4] Hegeman MA, Hennus MP, Heijnen CJ, Specht PA, Lachmann B, Jansen NJ (2009). Ventilator-induced endothelial activation and inflammation in the lung and distal organs. Crit Care.

[5] Klinger RY, Cooter M, Berger M (2016). Effect of intravenous lidocaine on the transcerebral inflammatory response during cardiac surgery: a randomized-controlled trial. Can J Anaesth.

[6] Samary CS, Pelosi P, Leme Silva P, Rocco PRM (2016). Immunomodulation after ischemic stroke: potential mechanisms and implications for therapy. Crit Care.

[7] Yew WP, Djukic ND, Jayaseelan JSP, Walker FR, Roos KAA, Chataway TK, Muyderman H, Sims NR. Early treatment with minocycline following stroke in rats improves functional recovery and differentially modifies responses of peri-infarct microglia and astrocytes. J Neuroinflammation. 2019;16(1):6. 10.1186/s12974-018-1379-y PMC6325745 30626393

[8] González-López Adrián, López-Alonso Inés, Aguirre Alina, Amado-Rodríguez Laura, Batalla-Solís Estefanía, Astudillo Aurora, Tomás-Zapico Cristina, Fueyo Antonio, dos Santos Claudia C., Talbot Konrad, Albaiceta Guillermo M. (2013). Mechanical Ventilation Triggers Hippocampal Apoptosis by Vagal and Dopaminergic Pathways. American Journal of Respiratory and Critical Care Medicine.

